# Late-Onset Femoroacetabular Impingement Syndrome Following Knee Arthroscopy in a Retrospective Cohort

**DOI:** 10.3390/jcm13226992

**Published:** 2024-11-20

**Authors:** Nicole D. Rynecki, Matthew T. Kingery, Rachel Roller, Emily Berzolla, Christopher A. Colasanti, Thomas Youm

**Affiliations:** 1 Department of Orthopedic Surgery, NYU Langone Orthopedic Hospital, 301 East 17th Street, New York, NY 10010, USA; nicole.rynecki@nyulangone.org (N.D.R.); matthew.kingery@nyulangone.org (M.T.K.); christopher.colasanti@nyulangone.org (C.A.C.); thomas.youm@nyulangone.org (T.Y.); 2 Department of Orthopedic Surgery, Stanford University, 430 Broadway Street, Redwood City, CA 94063, USA; rachel.roller@stanford.edu

**Keywords:** hip arthroscopy, knee arthroscopy, femoroacetabular impingement syndrome, biomechanics

## Abstract

**Background/Objectives:** Hip–knee coupling is a well-documented phenomenon, and interventions to one joint can alter biomechanics at the other. The purpose of this study was to investigate if knee surgery is associated with later onset of femoroacetabular impingement syndrome (FAIS). **Methods:** A retrospective chart review was conducted regarding patients at a single academic institution who underwent hip arthroscopy for FAIS between January 2011–October 2021. Patient charts were queried for past surgical history of knee arthroscopy before hip arthroscopy. Patients who previously underwent hip arthroscopy with no history of knee arthroscopy served as controls. Details about demographics and the onset of hip symptoms were abstracted from patient charts. Statistical analysis was conducted using Mann–Whitney testing and binary logistic regression. **Results:** Of the 1569 patients identified, 127 had a history of knee arthroscopy and reported no hip symptoms at or prior to the time of surgery. Patients who had undergone prior knee arthroscopy were significantly older at onset of initial hip symptoms (42.15 ± 11.80 years versus 34.62 ± 12.49 years, *p* < 0.001) and at the time of hip arthroscopy (44.12 ± 11.85 years versus 36.90 ± 12.14 years, *p* < 0.001) when controlling for age, sex, and BMI. These patients first developed hip symptoms at a mean of 8.57 ± 8.53 years following knee arthroscopy (median 6.10 years) and underwent operative treatment 1.76 ± 1.96 years later. **Conclusions:** Patients with a history of prior knee arthroscopy are older at the time of hip symptom onset and subsequent hip arthroscopy for the treatment of FAIS.

## 1. Introduction

Femoroacetabular impingement syndrome (FAIS) is due to osseous pathoanatomy of the femoral head and/or acetabulum, resulting in abnormal contact stresses [1]. Depending on whether the femoral head or acetabulum is the source of impingement, FAIS can be classified as cam or pincer, respectively, with many cases resulting from a combination of the two, thus appropriately denoted and combined. Cam morphology is femoral head asphericity and decreased head–neck offset. Radiographically, this is defined as an alpha angle > 55° [2]. Pincer deformity is acetabular overcoverage, radiographically seen in the setting of anterolateral overgrowth [lateral center edge angle (LCEA) > 40°], acetabular retroversion (crossover sign, ischial spine sign, and posterior wall sign), coxa profunda, or os acetabuli [2,3]. Both pathologies cause repetitive labral shearing and joint microtrauma, which ultimately can result in labral tears and chondral delamination [2,4]. It manifests clinically in patients as hip or groin pain during movements of flexion and internal rotation. It also is frequently associated with mechanical symptoms of clicking or catching [5]. 

Prior studies have estimated that approximately 10–24% of people meet radiographic criteria for femoracetabular impingement (FAI), reaching as high as 60% in some analyses of high-level athletes [6,7,8,9,10,11,12]. However, a vast majority of patients are asymptomatic, and prophylactic surgery is generally not recommended for these patients [6,12,13]. As such, there has been much interest in determining which patients with radiographic FAI become symptomatic and what risk factors lead to the clinical manifestations of FAIS.

The hip and knee have a complex neuromuscular relationship. Knee injuries are oftentimes the result of muscle weakness at the hip [14,15,16]. Thus, many knee injury prevention programs focus on hip abductor and external rotator strengthening and dynamic hip control due to their influence on landing mechanics at the knee [15,17]. Additionally, many knee injury rehabilitation and physical therapy programs, such as those for patellofemoral syndrome, can be successfully treated by targeting deficits at the hip [18,19]. Therefore, it is unsurprising that if interventions at one joint can improve biomechanics and alleviate pain at the other, the reverse is also true: interventions at one joint can have negative biomechanical implications for the other. Knee surgery, specifically ACL reconstructions and meniscectomies, have been shown to alter both joint loading and gait mechanics [20,21,22,23,24,25]. As our understanding of the hip–knee relationship continues to evolve and the use of hip arthroscopy to treat symptomatic FAIS expands, the purpose of this study is to investigate a relationship between prior knee surgery and the onset of FAIS. We hypothesized that knee arthroscopy, the most common knee surgery performed in the non-arthritic knee, would be associated with later-onset FAIS.

## 2. Materials and Methods

### 2.1. Study Design

A retrospective review of the electronic medical record (EMR) at a single, academic institution was queried for all patients who underwent hip arthroscopy for FAIS between January 2011 and October 2021. IRB approval was obtained (i20-01686).

### 2.2. Patient Selection

Patients were identified by current procedural terminology (CPT) codes for hip arthroscopy. These codes encompassed all procedures frequently performed for different FAIS pathology including femoroplasty (CPT-29914), acetabuloplasty (CPT-29915), and labral repair (CPT-29916). Patients were excluded if they had a history of slipped capital epiphysis (SCFE), Perthes disease, femoral neck stress fracture, or prior hip surgery before their knee arthroscopy or hip arthroscopy. Patients were also excluded if the indication for hip arthroscopy was for anything other than the treatment of FAIS (e.g., irrigation and debridement of a septic hip). With regards to knee surgery, included procedures were limited to partial meniscectomy, meniscal repair, chondroplasty, and arthroscopically assisted anterior crucial ligament (ACL) reconstruction. Patients undergoing arthroscopy in the setting of fracture fixation, any procedure involving an osteotomy, or arthroscopic treatment of infection were excluded. 

Charts were reviewed for a past surgical history of knee arthroscopy or arthroscopically assisted knee surgery that preceded hip arthroscopy. Patients who did not undergo relevant knee surgery prior to hip arthroscopy served as the control group. The control patients were selected from the same overall cohort as the knee arthroscopy group and were confirmed to have no ipsilateral procedures or hip injuries. Demographic information including age, sex, and body mass index (BMI), and details of the onset of hip symptoms, including quality and duration of symptoms, as described by patient history were collected from the EMR. Available knee and hip surgical characteristics including laterality and additional procedures performed (e.g., synovectomy and loose body removal) were also collected. 

Patients who underwent hip arthroscopy all had symptomatic FAIS. FAIS was diagnosed based on clinical symptoms (pain with flexion or rotation, catching or locking) and physical examination findings (limited range of motion, positive impingement tests) as well as corresponding radiographic and magnetic resonance imaging (MRI) findings. Radiographic findings indicative of FAIS included lateral center-edge angle ≥ 40°, alpha angle ≥ 55° on standing anteroposterior (AP), 45° and 90° Dunn views, as well as cox profunda, labral tears, and proximal focal acetabular retroversion on MRI.

### 2.3. Statistical Analysis

Analysis was performed using R version 4.1.1 (The R Foundation for Statistical Computing, Vienna, Austria). Standard descriptive statistics were used to evaluate the demographics of the cohort. Age at the onset of hip pain and age at the time of hip arthroscopy were compared between patients who had a history of knee surgery and patients without a history of knee surgery using *t*-tests. A multiple linear regression model was used to evaluate the effect of prior knee surgery on the development of hip symptoms when controlling for the effects of sex, BMI, and type of hip procedures performed.

## 3. Results

The initial query including any of the above CPT codes yielded 1569 patients. A total of 1474 patients who underwent arthroscopic treatment of FAIS during the study period were included in the final cohort, after excluding patients as described in the methods.

Among all patients included in the analysis, 59% were female, and the mean BMI was 25.65 ± 4.88. The mean age at the onset of hip symptoms was 35.61 ± 12.66 years and the mean age at the time of hip arthroscopy was 37.81 ± 12.34 years. In total, 97% of patients underwent labral repair, 31% underwent acetabuloplasty, and 28% underwent femoroplasty (Table 1).

**Figure 1:**
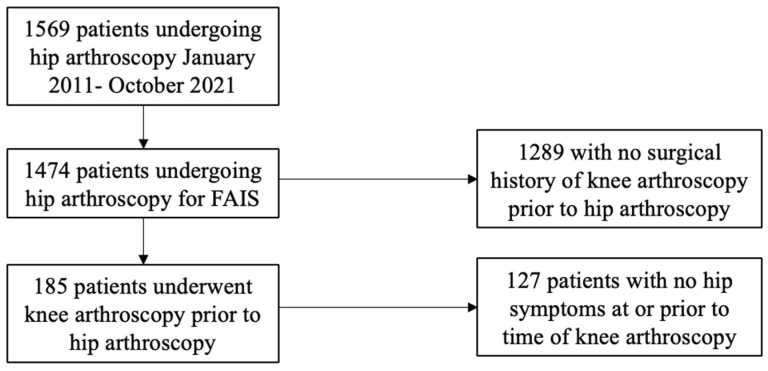
Patients meeting inclusion criteria, divided into two cohorts based on a surgical history of knee arthroscopy prior to hip arthroscopy.

In total, 12.55% of the overall cohort (185 patients) had undergone knee surgery prior to their hip arthroscopy (Figure 1). Thirty-four patients (18.38%) had undergone ACL reconstruction, while the remainder had undergone partial meniscectomy with or without chondroplasty. Of these patients, 50.27% subsequently underwent hip arthroscopy on the ipsilateral side of their prior knee surgery, and 35.68% underwent hip arthroscopy on the contralateral side. The laterality of the prior knee procedure was unknown for 26 patients (14.05%). 

**Figure 2:**
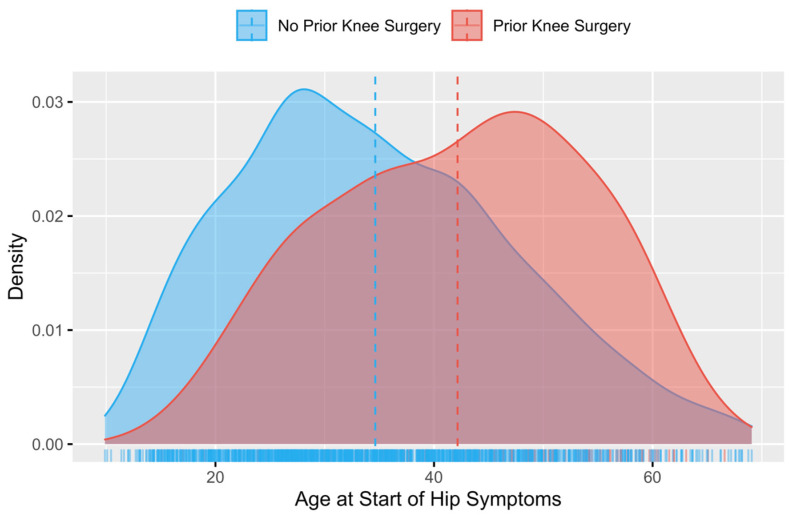
Age distribution of all patients undergoing hip arthroscopy for FAIS, demonstrating that patients with a past surgical history of knee arthroscopy were significantly older on presentation for hip symptoms.

When compared to patients who had no history of prior knee surgery, patients who had undergone prior knee surgery had a significantly greater age at the onset of initial hip symptoms (42.15 ± 11.80 years versus 34.62 ± 12.49 years, 95% CI [5.68, 9.38], *p* < 0.001; Figure 2). Similarly, patients who had previously undergone knee surgery were significantly older at the time of hip arthroscopy (44.12 ± 11.85 years versus 36.90 ± 12.14 years, 95% CI [5.38, 9.07], *p* < 0.001). Both patients who had knee surgery on the ipsilateral knee of their subsequent hip arthroscopy and patients who had knee surgery on the contralateral knee were significantly older at the time of onset of hip symptoms compared to patients without prior knee surgery (*p* < 0.001 for both comparisons). However, there was no significant difference in the age at which ipsilateral knee surgery patients developed hip symptoms versus the age at which contralateral knee surgery patients developed hip symptoms (43.29 ± 11.27 years versus 41.79 ± 12.70 years respectively, *p* = 0.421).

Patients who had previously undergone knee surgery had a significantly greater BMI at the time of hip arthroscopy compared to those without a history of significant knee pathology (27.70 ± 5.47 versus 25.33 ± 4.72, 95% CI [1.53, 3.20], *p* < 0.001). Given that BMI increased with increasing patient age in this cohort (0.09 kg/m^2^ per year based on linear regression), much of the difference in BMI between groups can be explained by the greater age of the group of patients with a history of prior knee surgery. When controlling for the effects of BMI, sex, and specific surgical procedures performed, patients with a history of prior knee surgery were found to develop hip symptoms 6.09 years after patients without prior knee surgery (95% CI [4.17, 8.01], *p* < 0.001). The surgical procedures performed (used as a proxy of underlying pathology, i.e., cam versus pincer versus combined FAIS) were not significantly associated with age at the onset of hip symptoms.

**Figure 3:**
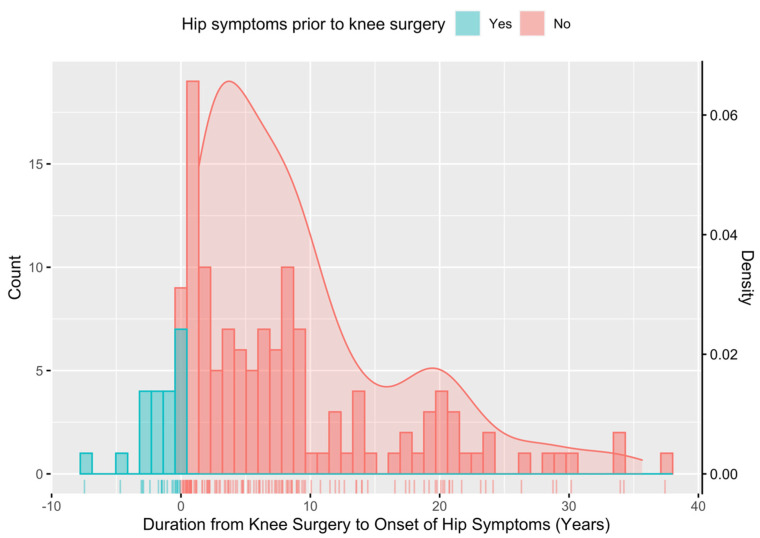
Chronology of the development of hip symptoms in relation to the date of knee arthroscopy, with 68.65% reporting no hip symptoms prior to or at the time of knee arthroscopy.

Among the patients with a history of knee surgery, 127 patients (68.65%) had no hip symptoms at (or prior to) the time of knee surgery. Patients in this specific group of interest first developed hip symptoms at a mean of 8.57 ± 8.53 years following the time of knee surgery (median 6.10 years) and then subsequently underwent operative treatment 1.76 ± 1.96 years later. Twenty-three patients (18.11%) who had no hip symptoms prior to knee surgery subsequently developed hip pain within 1 year of knee surgery (Figure 3).

## 4. Discussion

The most important finding from our study is that prior knee arthroscopy is associated with a delayed onset of hip symptoms and FAIS compared to patients with FAIS but no history of knee surgery. Patients were approximately seven and a half years older at the time of hip symptom onset compared to patients without a history of knee surgery. Subsequently, these patients were also significantly older at the time of hip arthroscopy for FAIS. The results of our sub-analysis of procedures performed during hip arthroscopy, used as a surrogate for type of FAIS, demonstrate that this trend was not specific to any one of the three FAIS pathologies (cam versus pincer versus combined). 

Identifying patients with later-onset FAIS is important because the literature demonstrates that older patients may have inferior outcomes. Horner et al. performed a systematic review of 17 studies of 16,327 patients, 9954 of which were older than 40, and found the rate of conversion to total hip arthroplasty (THA) was significantly higher in the older patients, who on average converted at approximately 2 years postoperatively. Specifically, they found a rate of conversion to THA of 18.1% in patients 40 and older and 23.1% in those older than 50 [26]. Allahabadi et al. [27] demonstrated that the risk of conversion to THA within 2 years after hip arthroscopy was increased significantly with age. In our investigation, patients with a history of knee surgery were on average 42 years old and underwent hip arthroscopy for FAIS at 44 years old. Given that the efficacy of hip arthroscopy may decrease with age based on current literature, every effort should be made to identify symptoms of FAIS in these patients earlier to allow for intervention at a younger age. Further, these patients should be appropriately counseled on postoperative expectations and the risk of conversion to THA. 

The association between knee arthroscopy and hip arthroscopy has not been reported in the literature. There are several possible mechanisms by which specific knee pathologies, such as ACL or meniscal tear, may be correlated with femoroacetabular impingement syndrome (FAIS). One potential explanation is that patients with knee pathology may have underlying biomechanical predispositions that make them more likely to develop hip pathology, and vice versa. Philippon et al. [28] demonstrated a 27-fold increased risk of ACL rupture in patients with alpha angles > 60° due to altered lower extremity biomechanics. Additionally, Beaulieu et al. [29] reported a 20% greater degree of ACL strain in patients for every 10° loss of internal femoral rotation, a deficit common in patients with FAIS. These findings suggest that patients with FAIS are more likely to have concomitant ACL injury, perhaps explaining the high incidence of knee arthroscopy prior to hip arthroscopy for FAIS. 

Another potential theory for a biomechanical link between these procedures is one of causality rather than correlation; perhaps procedures such as meniscectomy or ACL reconstruction (ACLR) alter hip alignment to create or worsen already-present FAIS. Wellsandt et al. [25] found that patients after ACL reconstruction ambulate with less external rotation. Internal rotation is a provocative movement for patients with FAIS, and thus this change alone could plausibly lead to new impingement symptoms. Numerous other studies have been published on lower extremity muscular deficits after ACL reconstruction and meniscectomy and their effects on gait and landing [24,30,31,32,33]. Deficits in muscles that cross both the hip and knee joint, such as the rectus femoris and the hamstrings, can ultimately lead to compensatory biomechanics and may be a pain generator at the hip joint. The later onset of hip symptoms among patients with a history of knee arthroscopy found in our study further supports the possibility that knee surgery can contribute to the development or worsening of FAIS symptoms via altered biomechanics. Given that knee arthroscopy may predispose patients to hip impingement, rehabilitation programs should consider targeted preventative strategies to mitigate the risk of FAIS.

### Limitations

While our study is among the first to investigate the relationship between knee surgery and delayed onset of FAIS, it is not without limitations. Given that this is a retrospective review of an institution’s EMR, we are limited by the documentation. For those patients who underwent knee arthroscopy at an outside institution, fewer details were available. Information regarding the knee arthroscopy was obtained via patient-reported surgical history and thus is subject to limitations of their health literacy and knowledge of the details of their prior surgery. Furthermore, our cohort likely underestimates the true incidence of knee surgery before hip arthroscopy, as patients were not captured if it was not reported in the surgical history. However, for patients who underwent knee arthroscopy at our institution, detailed surgical encounter information was available. Our specific cohort of interest is likely further underestimated, as older patients undergoing meniscectomies do not infrequently undergo total knee arthroplasty within several years, which excluded them from our study. Additionally, there was a percentage of patients for whom the exact onset of hip symptoms was not documented. This was particularly true for patients who had symptoms for “many years” and were unable to provide more detailed information, potentially biasing our time from hip symptom onset to hip arthroscopy towards a smaller time frame. However, this was the case for all patients included in the study and did not differ between cohorts, as all patients were indicated for hip arthroscopy due to hip symptoms. 

## 5. Conclusions

Patients with a history of prior knee arthroscopy are older at the time of hip symptom onset and subsequent hip arthroscopy for FAIS, and they also present with a higher BMI compared to those without prior knee surgery. 

## Figures and Tables

**Table 1:** Demographic and surgical characteristics of all patients who underwent hip arthroscopy for the treatment of FAIS during the study period.

Characteristic	N = 1474
Sex	
Female	877 (59%)
Male	597 (41%)
BMI	25.65 ± 4.88
Age at onset of hip symptoms (years)	35.61 ± 12.66
Age at time of hip arthroscopy (years)	37.81 ± 12.34
Duration from symptom onset to hip arthroscopy (years)	2.19 ± 2.53
Laterality of FAIS	
Left	674 (46%)
Right	800 (54%)
Procedures performed:	
Labral repair	1428 (97%)
Labral debridement	184 (12%)
Femoroplasty	420 (28%)
Acetabuloplasty	462 (31%)
Synovectomy	160 (11%)
Loose body removal	26 (1.8%)

## Data Availability

The data presented in this study are available on request from the corresponding author due to privacy concerns.

## References

[1] Trigg S.D., Schroeder J.D., Hulsopple C. (2020). Femoroacetabular Impingement Syndrome. Curr. Sports Med. Rep..

[2] Kapron A.L., Peters C.L., Aoki S.K., Beckmann J.T., Erickson J.A., Anderson M.B., Pelt C.E. (2015). The prevalence of radiographic findings of structural hip deformities in female collegiate athletes. Am. J. Sports Med..

[3] Direito-Santos B., França G., Nunes J., Costa A., Rodrigues E.B., Silva A.P., Varanda P. (2018). Acetabular retroversion: Diagnosis and treatment. EFORT Open Rev..

[4] Hoffer A.J., Beel W., Ng KC G., Degen R.M. (2024). The Contribution of Soft Tissue and Bony Stabilizers to the Hip Suction Seal: A Systematic Review of Biomechanical Studies. Am. J. Sports Med..

[5] Pun S., Kumar D., Lane N.E. (2015). Femoroacetabular impingement. Arthritis Rheumatol..

[6] Vahedi H., Aalirezaie A., Azboy I., Daryoush T., Shahi A., Parvizi J. (2019). Acetabular labral tears are common in asymptomatic contralateral hips with femoroacetabular impingement. Clin. Orthop. Relat. Res..

[7] Polat G., Şahin K., Arzu U., Kendirci A.Ş., Aşık M. (2018). Prevalence of asymptomatic femoroacetabular impingement in Turkey; cross sectional study. Acta Orthop. Et Traumatol. Turc..

[8] Hack K., Di Primio G., Rakhra K., Beaulé P.E. (2010). Prevalence of cam-type femoroacetabular impingement morphology in asymptomatic volunteers. JBJS.

[9] Reichenbach S., Jüni P., Werlen S., Nüesch E., Pfirrmann C.W., Trelle S., Odermatt A., Hofstetter W., Ganz R., Leunig M. (2010). Prevalence of cam-type deformity on hip magnetic resonance imaging in young males: A cross-sectional study. Arthritis Care Res..

[10] Mascarenhas V.V., Rego P., Dantas P., Morais F., McWilliams J., Collado D., Marques H., Gaspar A., Soldado F., Consciência J.G. (2016). Imaging prevalence of femoroacetabular impingement in symptomatic patients, athletes, and asymptomatic individuals: A systematic review. Eur. J. Radiol..

[11] Frank J.M., Harris J.D., Erickson B.J., Slikker I.I.I.W., Bush-Joseph C.A., Salata M.J., Nho S.J. (2015). Prevalence of femoroacetabular impingement imaging findings in asymptomatic volunteers: A systematic review. Arthrosc. J. Arthrosc. Relat. Surg..

[12] Diesel C.V., Ribeiro T.A., Scheidt R.B., de Souza Macedo C.A., Galia C.R. (2015). The prevalence of femoroacetabular impingement in radiographs of asymptomatic subjects: A cross-sectional study. Hip Int..

[13] Anzillotti G., Iacomella A., Grancagnolo M., Bertolino E.M., Marcacci M., Sconza C., Kon E., Di Matteo B. (2022). Conservative vs. Surgical Management for Femoro-Acetabular Impingement: A Systematic Review of Clinical Evidence. J. Clin. Med..

[14] Nadler S.F., Malanga G.A., DePrince M., Stitik T.P., Feinberg J.H. (2000). The relationship between lower extremity injury, low back pain, and hip muscle strength in male and female collegiate athletes. Clin. J. Sport Med..

[15] Lopes TJ A., Simic M., Myer G.D., Ford K.R., Hewett T.E., Pappas E. (2018). The Effects of Injury Prevention Programs on the Biomechanics of Landing Tasks: A Systematic Review With Meta-analysis. Am. J. Sports Med..

[16] Mucha M.D., Caldwell W., Schlueter E.L., Walters C., Hassen A. (2017). Hip abductor strength and lower extremity running related injury in distance runners: A systematic review. J. Sci. Med. Sport.

[17] Malloy P.J., Morgan A.M., Meinerz C.M., Geiser C.F., Kipp K. (2016). Hip External Rotator Strength Is Associated With Better Dynamic Control of the Lower Extremity During Landing Tasks. J. Strength Cond. Res..

[18] Santos T.R., Oliveira B.A., Ocarino J.M., Holt K.G., Fonseca S.T. (2015). Effectiveness of hip muscle strengthening in patellofemoral pain syndrome patients: A systematic review. Braz. J. Phys. Ther..

[19] Ferber R., Bolgla L., Earl-Boehm J.E., Emery C., Hamstra-Wright K. (2015). Strengthening of the hip and core versus knee muscles for the treatment of patellofemoral pain: A multicenter randomized controlled trial. J. Athl. Train..

[20] Ferber R., Kendall K.D., Farr L. (2011). Changes in knee biomechanics after a hip-abductor strengthening protocol for runners with patellofemoral pain syndrome. J. Athl. Train..

[21] Boggess G., Morgan K., Johnson D., Ireland M.L., Reinbolt J.A., Noehren B. (2018). Neuromuscular compensatory strategies at the trunk and lower limb are not resolved following an ACL reconstruction. Gait Posture.

[22] Thorlund J.B., Holsgaard-Larsen A., Creaby M.W., Jørgensen G.M., Nissen N., Englund M., Lohmander L.S. (2016). Changes in knee joint load indices from before to 12 months after arthroscopic partial meniscectomy: A prospective cohort study. Osteoarthr. Cartil..

[23] Karahan M., Özcan M., Cığalı B.S. (2022). Balance Evaluation and Gait Analysis After Arthroscopic Partial Meniscectomy. Indian J. Orthop..

[24] Sugimoto D., Heyworth B.E., Yates B.A., Kramer D.E., Kocher M.S., Micheli L.J. (2019). Effect of graft type on thigh circumference, knee range of motion, and lower-extremity strength in pediatric and adolescent males following anterior cruciate ligament reconstruction. J. Sport Rehabil..

[25] Wellsandt E., Zeni J., Axe M., Snyder-Mackler L. (2017). Hip joint biomechanics in those with and without post-traumatic knee osteoarthritis after anterior cruciate ligament injury. Clin. Biomech..

[26] Horner N.S., Ekhtiari S., Simunovic N., Safran M.R., Philippon M.J., Ayeni O.R. (2017). Hip arthroscopy in patients age 40 or older: A systematic review. Arthrosc. J. Arthrosc. Relat. Surg..

[27] Allahabadi S., Hinman A.D., Horton B.H., Avins A.L., Coughlan M.J., Ding D.Y. (2020). Risk factors for conversion of hip arthroscopy to total hip arthroplasty: A large closed-cohort study. Arthrosc. Sports Med. Rehabil..

[28] Philippon M., Dewing C., Briggs K., Steadman J.R. (2012). Decreased femoral head–neck offset: A possible risk factor for ACL injury. Knee Surg. Sports Traumatol. Arthrosc..

[29] Beaulieu M.L., Oh Y.K., Bedi A., Ashton-Miller J.A., Wojtys E.M. (2014). Does limited internal femoral rotation increase peak anterior cruciate ligament strain during a simulated pivot landing?. Am. J. Sports Med..

[30] Casartelli N.C., Item-Glatthorn J.F., Friesenbichler B., Bizzini M., Salzmann G.M., Maffiuletti N.A. (2019). Quadriceps neuromuscular impairments after arthroscopic knee surgery: Comparison between procedures. J. Clin. Med..

[31] Mesnard G., Fournier G., Joseph L., Shatrov J.G., Lustig S., Servien E. (2022). Does meniscal repair impact muscle strength following ACL reconstruction?. SICOT—J..

[32] Ericsson Y.B., Roos E.M., Owman H., Dahlberg L.E. (2019). Association between thigh muscle strength four years after partial meniscectomy and radiographic features of osteoarthritis 11 years later. BMC Musculoskelet. Disord..

[33] Bruce Leicht A.S., Thompson X.D., Kaur M., Hopper H.M., Stolzenfeld R.L., Wahl A.J., Sroufe M.D., Werner B.C., Diduch D.R., Gwathmey F.W. (2023). Hip Strength Recovery After Anterior Cruciate Ligament Reconstruction. Orthop. J. Sports Med..

